# Nanostructured carbon black for simultaneous electrochemical determination of trace lead and cadmium by differential pulse stripping voltammetry

**DOI:** 10.1098/rsos.180282

**Published:** 2018-07-25

**Authors:** Ruigang Xie, Lingli Zhou, Cuiling Lan, Fangfang Fan, Ruifeng Xie, Hongyu Tan, Tiansheng Xie, Lingmin Zhao

**Affiliations:** 1 Guangxi Colleges and Universities Key Laboratory of Regional Ecological Environment Analysis and Pollution Control of West Guangxi, College of Chemistry and Environment Engineering, Baise University, Baise 533000, People's Republic of China; 2 Key Laboratory of New Processing Technology for Nonferrous Metals and Materials, Ministry of Education, College of Materials Science and Engineering, Guilin University of Technology, Guilin 541004, People's Republic of China; 3 School of Naval Architecture, Ocean and Civil Engineering, Shanghai Jiao Tong University, Shanghai 200240, People's Republic of China; 4 Guangxi Ferroalloy Co. Ltd, Laibin 546102, People's Republic of China

**Keywords:** carbon nanomaterials, electrochemical determination, stripping voltammetry

## Abstract

Nanostructured carbon black (CB) was first employed directly in this paper for the simultaneous electrochemical determination of trace Pb(II) and Cd(II) using differential pulse anodic stripping voltammetry. The morphology and surface properties of conductive CB were characterized by transmission electron microscopy, X-ray diffraction, X-ray photoelectron spectroscopy, ultraviolet–visible spectroscopy and Raman spectroscopy. Special pore structures, as well as surface chemical functional groups, endow CB with excellent catalytic and adsorption properties. Some parameters affecting electrical analysis performance were investigated systematically including deposition time and potential, pH value of solution, volume of suspension, amount of Bi(III) and Nafion solution. CB–Nafion–glassy carbon electrode sensor linear response ranges from 6 to 1000 nM for selective and simultaneous determination. The detection limits were calculated to be 8 nM (0.9 µg l^−1^) for Cd(II) and 5 nM (1.0 µg l^−1^) for Pb(II) (S/N = 3) for the electrocatalytic determination under optimized conditions. The method was successfully used to the determination of actual samples and good recovery was achieved from different spiked samples. Low detection limits and good stability of the modified electrode demonstrated a promising perspective for the detection of trace metal ions in practical application.

## 1. Introduction

Generally, heavy metal ions coming from industrial processes will endanger human health and the environment if discharged into nature without treatment. Among them, Pb(II) and Cd(II) are highly toxic and may lead to adverse effects on immune, central nervous and reproductive systems even at trace level. Hence, the research on detection of heavy metal ions focuses predominantly on detection technologies. Compared with detection methods such as atomic absorption/emission spectroscopy, inductively coupled plasma mass spectroscopy [1], atomic fluorescence [2,3], and colorimetric detection [4–6], electrochemical detection has been proven to be a quick, cost-effective method and suitable for online monitoring.

In recent years, various of materials have been explored to be an ideal sensor for applications in detection of heavy metal ions, including mesoporous silica nanoparticles [7], microporous carbon [8], carbon nanotubes [9,10], metal nanoparticles [11–13] and metal–organic frameworks [14]. Owing to the wide window of potential and high electrical conductivity, these modified electrodes achieve lower detection limits and broader test ranges in electrochemical analysis of lead and cadmium. Nevertheless, high prices and complicated preparation process of the modified working materials are the main drawbacks which may seriously affect the practical application in electrochemical detection of heavy metals.

Carbon black (CB) has been widely used for rubber or polymer reinforcement in many industrial applications due to its large surface area, nanoscale particles, abundant pore structure and high electrical conductivity. With low crystallinity and abundant active edges, CB can have higher catalytic activity than graphene and carbon nanotubes [15]. In the field of catalysis, CB is commonly chosen as a catalyst support in order to disperse and reduce metal nanoparticle agglomeration with high electrical conductivity and high stability [16,17]. In addition, CB has been successfully applied for detection of ferricyanide, benzoquinone, epinephrine, nitrite, nitrate and H_2_O_2_ in recent years [18,19]. However, simultaneous detection of Pb(II) and Cd(II) at CB-modified electrodes has not been reported so far. In terms of material stability, research on CB has a long history and mature technology. Compared with the carbon materials prepared by carbonization of other materials [13,20,21], classical CB is universal and stable in the application of modified electrode materials particularly. Thus, it is highly desirable to develop a facile and cost-effective method for the detection of toxic metal ions in terms of a practical point of view as compared with expensive materials and other complex preparation processes.

Anodic stripping voltammetry (ASV) is one of the most effective and robust means of electrochemical determination of trace heavy metal ions because of the characteristics of high sensitivity, good selectivity and low detection limits, relatively low cost, ability to simultaneously determine multiple elements, suitability for on-site analysis and *in situ* detection, simplicity and portability [22–24]. ASV detection process typically combines a deposition step and an electrochemical stripping step that provide a favourable signal to background ratio by pulsing the potential. Therefore, electrode materials are important to ASV measurement.

We report here a facile and cost-effective method for the fabrication of a CB-modified glassy carbon electrode (GCE) for application as a high-performance electrochemical sensor for simultaneous detection of trace lead and cadmium based on differential pulse stripping voltammetry. Some of the key parameters in the electrochemical analysis were investigated. Additionally, the anti-interference ability, reproducibility and repeatability were also investigated. Finally, the proposed CB-modified electrode was used to analyse Cd(II) and Pb(II) levels in real samples. In a typical process, conductive CB (XC-72) and Nafion (5 wt% solution of water and lower aliphatic alcohols) were mixed together in absolute ethanol to get a black coating solution. A drop of the prepared solution was placed onto the GCE surface. The simultaneous electrochemical determination of trace Pb and Cd was conducted with three electrodes in a 0.1 M acetate buffer (pH 4.5).

## 2. Material and methods

### 2.1. Reagents and materials

CB (Vulcan XC-72) was purchased from Cobot Co., Ltd. Nitric acid (65–68%), Nafion (perfluorosulfonic acid–polytetrafluoroethylene copolymer, 5% w/w) solution was obtained from DuPont. HAc, Cd(NO_3_)_2_·4H_2_O, Pb(NO_3_)_2_·9H_2_O, Bi(NO_3_)_3_·5H_2_O and NaAc were purchased from Sino pharm Group Chemical Reagent Co., Ltd. Ultra-pure water (18.2 MΩ cm) was used from a Milli-Q system. All the chemicals were used as received without further purification.

### 2.2. Fabrication of the CB–Nafion-modified electrode

Three-electrode system was used to perform electrochemical experiments with a CHI660e electrochemical workstation. GCE (3 mm), Ag/AgCl electrode and platinum wire acted as the working electrode, the reference electrode and the counter electrode, respectively.

CB (5 mg) was dispersed in anhydrous ethanol (2 ml, ≥ 99.7) with ultrasonication for 30 min and allowed to stand for another 10 min. Nafion solution ranging from 25 to 250 µl was injected to the above solution through microsyringe. 2.5 mg ml^−1^ CB–Nafion suspension was obtained. Bare GCE was polished with 0.05 mm alumina slurries until a mirror-like surface was obtained before modification. The prepared CB–Nafion suspension (3 µl) was dropped on the GCE surface and allowed to dry at room temperature.

### 2.3. Simultaneous detection of lead(II) and cadmium(II)

Differential pulse anodic stripping voltammetry (DPASV) was used for the simultaneous determination of lead(II) and cadmium(II) with an *in situ* deposition of bismuth film. The three electrodes were immersed into an electrochemical cell containing 10 ml of 0.1 mol l^−1^ acetate buffer solution (pH 5.0). The deposition potential of −1.1 V (versus Ag/AgCl) was used in the preconcentration step for 300 s under stirring. ASV was conducted without stirring at potentials ranging from −1.0 to 0 V. Prior to the next cycle, the metal deposited on the electrode surface was removed by electrolysis at 0.3 V for 30 s under stirring.

### 2.4. Apparatus

The morphology of the CB was obtained by a transmission electron microscopy (TEM) instrument (FEI Tecnai G2 F20, FEI, USA) operated at 200 kV. The powder wide-angle X-ray diffraction pattern (XRD) was recorded with an Ultima IV diffractometer (Rigaku, Japan). UV–visible absorption spectra were obtained with a Nicolet iz10 spectrophotometer (Thermo Scientific, USA). Raman spectra were obtained with an RM2000 (Renishaw, UK). X-ray photoelectron spectroscopy (XPS) was performed by an Esca lab 250Xi (Thermo Scientific, USA).

## 3. Results and discussion

### 3.1. Characterization studies

**Figure 1.**
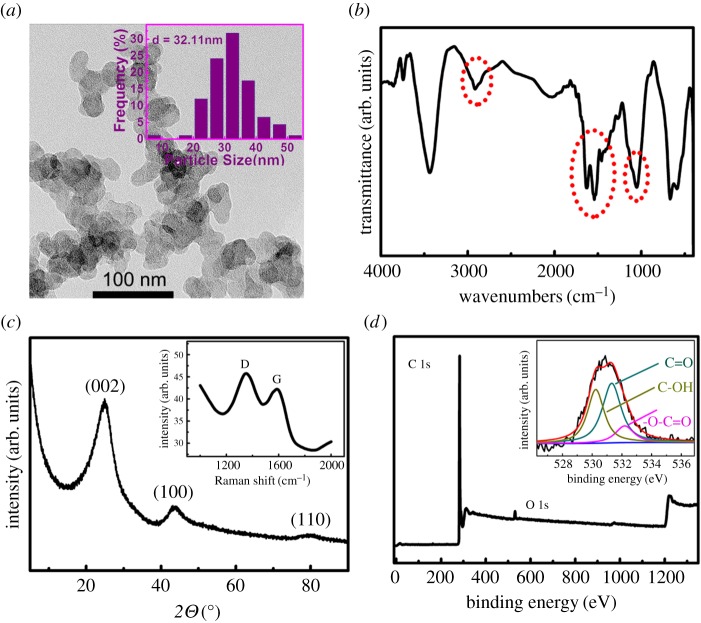
(*a*) TEM image of CB. (*b*) FTIR spectrum of CB. (*c*) XRD pattern of CB, the inset shows the Raman spectrum of CB. (*d*) XPS spectrum of CB, the inset shows XPS spectra of the O 1 s peak.

Figure 1*a* presents a typical TEM image of CB; the average size of CB was around 32 nm. CB particles formed together into ‘soap bubbles’ shaped aggregates with developed branches and pore structure, which is an advantage to the formation of conductive channels in the application. The Fourier transform infrared (FTIR) spectrum of CB in the range of 4000–400 cm^−1^ is shown in figure 1*b*. The intense broad peak at 2900 cm^−1^ corresponded with the carboxylic group (COOH). The absorption band between 1700 and 1400 cm^−1^ was attributed to the stretching vibration of C=O. The absorption bands at around 1078 and 1027 cm^−1^ were derived from C–O stretching vibration from ether and alcohol, respectively. It suggests that some COOH groups exist at the CB surface. Figure 1*c* presents the XRD pattern of CB; three peaks at 24.71, 43.75 and 79.84 were obviously observed and were attributed to (002), (100) and (110), respectively. The (002) diffraction peak corresponding to the hexagonal structure of graphitic structure of XC-72 carbon demonstrated that XC-72 carbon was well-graphitized. The Raman spectrum (figure 1*c* inset) of CB showed a higher G-band at 1585.99 cm^−1^ and a lower D-band at 1349.59 cm^−1^, demonstrating the high degrees of graphitization of the CB nanoparticles. The D-band indicated the existence of defects in the surface and edge of CB, and the relative intensity ratio between D-band and G-band (ID/IG = 1.25) is associated with the number of defect sites in graphite carbon [25]. XPS technique was used to indicate the surface functional groups of XC-72 carbon. Figure 1*d* presents the XPS spectra of XC-72 carbon. The relative peak intensity (figure 1*d* inset) at around 531 eV is related to O 1 s. The content of oxygen and carbon was 0.97 and 99.03. In order to further characterize the surface of XC-72 carbon, the O 1 s binding energy peaks of XC-72 carbon were consistent with three individual component peaks [26]: (i) the peak at 530.35 eV was attributed to carboxyl functional groups (–CO–O–); (ii) the peak at 531.58 eV was attributed to carbonyl functional groups (C=O); and (iii) the peak at 532.98 eV was attributed to hydroxyl functional groups (C–OH). These oxygen-containing groups can easily coordinate with Cd(II) and Pb(II). Thus, the deposition amount of Cd(II) and Pb(II) on the electrode surface can be increased.

### 3.2. Electrochemical properties

**Figure 2.**
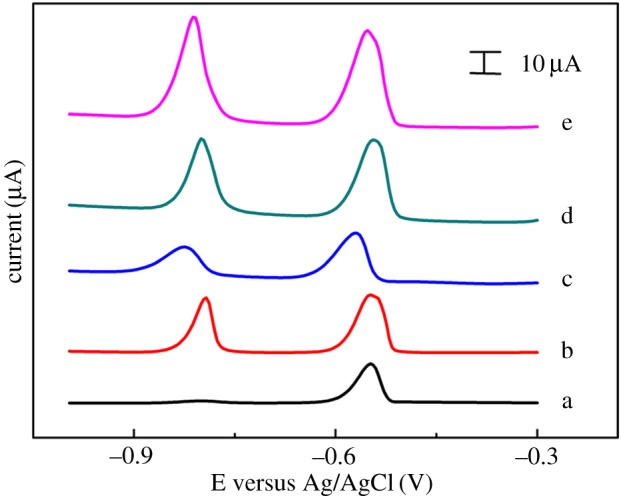
DPASV curves of 0.5 µM Cd(II) and Pb(II) obtained in 0.1 M HAc–NaAc buffer (pH = 4.5) at different electrodes: (a) bare GCE, (b) bare GCE in the presence of 5 µM Bi(III) ion, (c) CB–GCE, (d) CB–GCE in the presence of 5 µM Bi(III) ion and (e) CB–Nafion–GCE in the presence of 5 µM Bi(III) ion and 7.5% (v/v) Nafion solution in the CB suspension. Measurement conditions are the following: deposition potential −1.1 V, deposition time 300 s, amplitude 50 mV, pulse width 50 ms, potential step 4 mV.

Figure 2 presents the peak currents of 0.5 µM Cd(II) and 0.5 µM Pb(II) on different modified electrodes. As shown, the stripping response of Cd(II) at bare GCE was unobvious and the stripping response of Pb(II) at the bare GCE was weak (curve a). When Bi(III) ion was used for *in situ* formation of bismuth film (curve b) in the tested solution or CB was modified to the surface of the GCE (curve c), both Cd(II) and Pb(II) could be measured simultaneously. At the same time, the current signals of both ions were enhanced when CB-modified GCE was used in the presence of Bi(III) ion (curve d). Nafion was used to improve the stability of the test in the process of sample preparation. Electrochemical performance response value of both ions largely increased when Nafion was used to stabilize material modified on the electrode. Therefore, CB–Nafion–GCE was chosen for detection of Cd(II) and Pb(II) concentration simultaneously with *in situ* formations of bismuth film.

**Figure 3.**
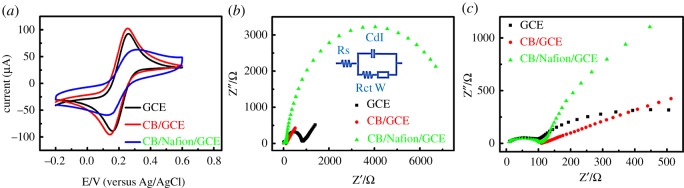
(*a*) Cyclic voltammetry diagrams at bare GCE, CB-GCE and CB–Nafion–GCE in the solution of 5 mM ferro-/ferricyanide containing 0.1 M KCl, scan rate: 100 mV s^−1^, (*b*) electrochemical impedance spectra of GCE, CB-GCE and CB–Nafion–GCE in the solution of 5 mM ferro-/ferricyanide containing 0.1 M KNO_3_ with a frequency range of 0.1–100 Hz, a bias potential of 0.2 V versus Ag/AgCl and an alternating current amplitude of 5 mV. The inset shows the Randle's equivalent circuit compatible with the electrochemical impedance diagram for this system. (*c*) Amplification of high-frequency section for impedance diagram of three kinds of electrodes.

Cyclic voltammetry (CV) and electrochemical impedance spectroscopy (EIS) were used to investigate the characteristics of different modified electrodes (bare GCE, CB–GCE and CB–Nafion–GCE). CV was carried out in 5.0 mM [Fe(CN)_6_]^3−/4−^ (1 : 1) solution containing 0.1 M KCl for different modified electrodes, shown in figure 3*a*. A clear and enhanced redox peak was obtained on CB–GCE, indicating that CB has good electron-transfer ability. However, CB–Nafion–GCE showed relatively lower redox current signals of [Fe(CN)_6_]^3−/4−^ compared with the bare GCE, which was due to the poor electrical conductivity of Nafion. Besides, EIS of [Fe(CN)6]^3−/4−^ was used to provide information in the frequency range from 1 to 10^5^ Hz at a potential of 0.2 V about the interface properties and impedance changes in the process of electrode modification. To the best of our knowledge, semicircle in Nyquist plot at high-frequency section was associated with electron-transfer resistance (Rct); moreover, the linear portion at lower frequencies presented the diffusion process. Figure 3*b* shows the Nyquist diagrams of different electrodes in 5.0 mM [Fe(CN)6]^3−/4−^ containing 0.1 M KCl. The equivalent circuit diagram was used to fit EIS data as shown in figure 3*b*. In the enlarged Nyquist diagram at higher frequencies (figure 3*c*), the Rct value of CB–GCE (136Ω) was smaller than that of the bare GCE (752Ω) due to the fact that the electronic conductivity of conductive CB improves the diffusion of ferricyanide to the electrode surface. A larger semicircle was observed in the same enlarged figure 3*c* at CB–Nafion–GCE compared with the bare GCE, indicating large interface electron-transfer impedance of CB–Nafion–GCE. This result is consistent with the conclusion of the CV test due to the nature of Nafion. However, Nafion in the solution can effectively disperse and combine with CB particles to form a relatively stable solution, thus improving its analytical ability.

### 3.3. Optimization of experimental conditions

**Figure 4.**
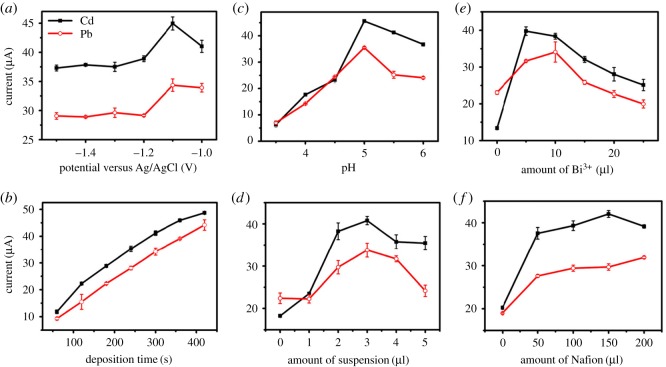
The effect of experimental parameters on the DPASV peak currents of 0.5 µM Cd(II) and Pb(II) in 0.1 M HAc–NaAc buffer. (*a*) Preconcentration potential, (*b*) deposition time, (*c*) pH value, (*d*) amount of suspension, (*e*) Bi(III) concentration and (*f*) Nafion concentration.

Some parameters involved with experiments, such as deposition time, deposition potential, pH value of solution, volume of suspension, amount of Bi(III) and Nafion solution, were investigated systematically for good performance stripping analysis of both metal ions. Figure 4 shows the effect of these parameters under the conditions of 0.5 µM Cd(II) and Pb(II). As the potential plays an important role in the sensitivity of ASV measurement, accumulation potentials from −1.5 to −1.0 V (versus Ag/AgCl) were investigated as shown in figure 4*a*. The peak current increased with deposition potential at first and then decreased. The maximal peak current was observed at −1.1 V both for Cd(II) and Pb(II). At the more negative potential, there will be hydrogen evolution, and the ions to be measured at the more negative potential will be greater interference [8]. Taking into account the above reasons, −1.1 V was chosen as accumulation potential for other parameters for further determination. Figure 4*b* illustrates the influence of the deposition time on the stripping currents of Cd(II) and Pb(II). For both metal ions, the current density at CB–Nafion–GCE increased linearly with deposition time from 60 to 420 s. With the deposition time prolonged, the linear trend becomes insignificant due to the saturated load on the electrode surface [7]. In this study, the deposition time of 300 s is suitable for the analysis of the standard solution and was then fully selected for all subsequent work.

The effect of pH on the DPASV response was studied from pH 3.5 to 6.0, and the results are shown in figure 4*c*. When the pH value increased from 3.5 to 5.0, the peak current increased significantly, reached its maximum at pH 5.0, and then decreased at pH 5.5. On the one hand, the electrostatic repulsion between the oxygen-containing groups of the CB–Nafion and Cd(II) and Pb(II) led to a low response at low pH value. On the other hand, the hydrolysis of the metal ions reduced current density [27]. From this point of view, pH 5.0 was selected for further studies. The effect of the volume of CB–Nafion suspension on the stripping peak current was obtained as shown in figure 4*d*. A suitable amount of 3 µl CB–Nafion modified on the electrode was optimized and used in the following experiments.

Compared with mercury-based electrodes, bismuth-based electrodes have lower toxicity and better performance due to the alloy with other tested metal ions. However, thick bismuth film might inhibit the mass transfer of the tested metal ions during the stripping step [28]. From the data in figure 4*e*, the suitable amount was 5 µl of 5 µM Bi(III) under the existing optimized parameters. Nafion solution was used in the determination of metal ions due to its cationic exchange capacity. The relationship between the amount of Nafion and the stripping signal was studied in the range of 0–200 µl as shown in figure 4*f*. The maximum peak current of Cd(II) and Pb(II) was obtained when 150 µl Nafion solution was selected in the process of preparing the samples to avoid sacrificing too much sensitivity for the simultaneous determination of Cd(II) and Pb(II) [8].

### 3.4. Stripping voltammograms and a calibration curve

**Figure 5.**
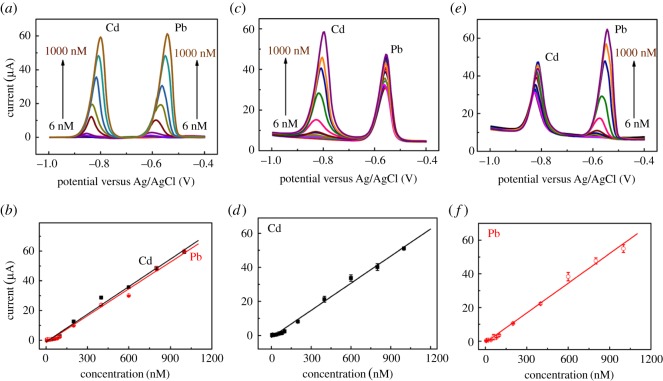
DPASV and calibration curves of the stripping peak currents at CB–Nafion–GCE with increased concentrations of Cd(II) and Pb(II) (*a,b*); increased concentrations of Cd(II) in the presence of 0.5 µM Pb(II) (*c,d*); increased concentrations of Pb(II) in the presence of 0.5 µM Cd(II) (*e,f*) in 0.1 M HAc–NaAc buffer solution. Other experimental conditions were under the optimal conditions.

The stripping voltammograms for different concentrations of Cd(II) and Pb(II) at the CB–Nafion–GCE are shown in figure 5*a,b*. The analytical performance of the proposed method for both metal ions determined simultaneously was evaluated under optimal conditions. From 6 to 1000 nM both for Cd(II) and Pb(II), the regression equation of the calibration curve is I*_p_* (μA) = 0.0619*C*_Cd_ (nM) − 1.094 (*R*^2^ = 0.991) for Cd(II) and I*_p_* (μA) = 0.0604*C*_Pb_ (nM) − 1.646 (*R*^2^ = 0.991) for Pb(II). The limit of detection (LOD) was calculated from 3SD/*S* (SD is the standard deviation of 0.1 µM Cd(II) and Pb(II) measurements (*n* = 11) and *S* is the slope of calibration curve). LODs were found to be 8 nM (0.9 µg l^−1^) for Cd(II) and 5 nM (1.0 µg l^−1^) for Pb(II), which are 3 times and 10 times lower than the safety values set by the World Health Organization in drinking water. Thus, the electrodes proposed in this work are sufficiently sensitive for the determination of Cd(II) and Pb(II) in water.

Selective determination of Cd(II) or Pb(II) was also applied to investigate the effects of ions as one ion concentration changed from 6 to 1000 nM while the other was maintained at a fixed concentration of 0.5 µM. As shown in figure 5*c,d*, two sets of sharp peaks corresponding to Cd(II) and Pb(II) appeared at about −0.82 V and −0.56 V, respectively. The stripping peak of Cd(II) shifted slightly towards positive direction as the concentration of Cd(II) increased and this trend applies equally to Pb(II) ions to be tested. With the increase of Cd(II), the peak current of Cd(II) increased significantly while the amount of Pb(II) remained unchanged. The calibration curve between the stripping current of Cd(II) and its concentration was fitted well to a linear equation I*_p_* (μA) = 0.0531*C*_Cd_ (nM) − 1.205 (*R*^2^ = 0.993) in a concentration range from 6 to 1000 nM as shown in figure 5*d*. Likewise, selective determination of Pb(II) in a fixed concentration of 0.5 µM Cd(II) was also performed as shown in figure 5*e,f*. The calibration curve for Pb(II) also shows good linearity over the concentration range of 6–1000 nM. The correlation equation for the calibration curve can be defined as I*_p_* (μA) = 0.0588*C*_Pb_ (nM) − 0.848 (*R*^2^ = 0.992). The proposed working electrode has a relatively lower detection line and a wider linear range than many other working electrodes summarized in table 1 for the electrochemical detection of Cd(II) and Pb(II), which may be attributed to the unique conductivity and adsorption properties of conductive CB.

**Table 1.** Comparison of analytical performance of differently modified electrodes.^a^

electrode type	detected metal	analysis method	LOD (μg l^−1^)	linear range (μg l^−1^)	accumulation time	references
nanoSiO_2_/BiFE	Pb	SWASV	Pb: 0.2	Pb: 2–150	120	[7]
	Cd		Cd: 0.6	Cd: 2–150	120	
Nafion/Bi/NMC/GCE	Pb	DPASV	Pb: 0.05	Pb: 0.5–10/10–100	150	[8]
	Cd		Cd: 1.5	Cd: 2–10/10–100	150	
MWNTs/Nafion film	Pb	DPV	Pb: 4.1	Pb: 17–1240	120	[9]
engineered MWCNTs	Pb	SWASV	Pb: 0.3	Pb: 2–50	400	[10]
	Cd		Cd: 0.4	Cd: 2–50		
Au NPs/GCE	Cd	DPASV	—	Pb: 62–290	300	[11]
	Pb		—	Cd: 34–157	300	
	Cu		—	Cu: 19–89	300	
	Hg		—	Hg: 60–280	300	
GNP/PANI/GR/GCE	Pb	DPASV	Pb: 0.02	Pb: 0.1–2.1	240	[12]
Pd/PAC/GCE	Cd	DPV	Pb: 10.4	Pb: 103–1842	—	[13]
	Pb		Cd: 4.6	Cd: 56–616	—	
	Cu		Cu: 4.2	Cu: 32–317	—	
	Hg		Hg: 10.8	Hg: 48–1504	—	
MIL-101(Cr)/GCE	Pb	DPASV	Pb: 0.1	Pb: 0.2–207	600	[14]
CB–Nafion–GCE	Pb	DPASV	Pb: 1.0	Pb: 1.2–207	300	this study
	Cd		Cd: 0.9	Cd: 0.6–112	300	this study

^a^SWASV, square wave stripping voltammetry; DPASV, differential pulse anodic stripping voltammetry; DPV, differential pulse voltammetry.

### 3.5. The interference study

The interference study was conducted by adding various inorganic species into solution containing 0.5 µM each of Cd(II) and Pb(II) under optimized conditions. Table 2 suggests that a great number of cations, such as Ca(II), Zn(II), Al(III), K(I), Mg(II), Ba(II) and Fe(III), have little influence on the signals of Cd(II) and Pb(II) (each 50 µM), with the deviation below 9%. However, Cu(II) was found to interfere with the determination of Cd(II) and Pb(II) drastically, which may be attributed to the intermetallic compounds formed by interfering ions and heavy metal ions [29]. We found that the addition of 20 mM potassium ferrocyanide could reduce the interference of copper ions with a concentration of 10 times higher than that of lead and cadmium.

**Table 2.** Interferences of some metal ions on the stripping peak currents of 0.5 µM Pb(II) and Cd(II).

		relative signal change (%)	
interferences	concentration (μM)	Cd(II)	Pb(II)
K(I)	50	−8.23	−2.72
Ba(II)	50	2.98	0.34
Ca(II)	50	−0.24	−6.01
Al(III)	50	−8.51	−5.79
Zn(II)	50	−4.66	−8.06
Mg(II)	50	−6.22	−5.82
Fe(III)	25	−7.76	−5.32
Cu(II)	1	76.17	−44.99

### 3.6. Repeatability, reproducibility and stability of the proposed sensor

Repeatability of the CB-modified sensor was evaluated by parallel detecting 0.5 µM Cd(II) and Pb(II) for 11 times. The relative standard deviations were 4.48% for Cd(II) and 6.06% for Pb(II). Six independent modified electrodes were used to investigate the reproducibility of the electrodes in the Cd(II) and Pb(II) solution (0.5 µM). The peak current relative standard deviations of Cd(II) and Pb(II) were 3.13% and 4.29%, respectively. In addition, the peak currents retained 93% and 95% of the original response after the electrode was stored for 10 days at 4°C. These results indicated that CB-modified sensor exhibited good repeatability, reproducibility and stability.

### 3.7. Analytical performances of real samples

The proposed method was applied to determine Pb(II) in real water samples from tap water, Baicheng purified water and Youjiang river water for determining the accuracy of this method. The sample was filtered with a 0.24 µm Millipore membrane and diluted with 0.1 M acetate buffer (pH 4.5) in a ratio of 2 : 8. The DPASVs were recorded before and after spiking with Cd(II) and Pb(II) standard solutions to samples. The results after analysis for Cd(II) and Pb(II) are shown in table 3. It was confirmed that the determination of cadmium and lead ions at CB–Nafion–GCE was not affected by sample matrix.

**Table 3.** Determination of Cd(II) and Pb(II) in different samples (each determination was carried out for three times).

		found (μM)		Recovery (%)	
sample	spiked (μM)	Cd(II)	Pb(II)	Cd(II)	Pb(II)
tap water	0	—	—	—	—
	0.30	0.325	0.287	108	95.7
	0.50	0.521	0.483	104	96.6
purified water	0	—	—	—	—
	0.30	0.312	0.293	104	97.6
	0.50	0.505	0.494	101	98.8
Youjiang water	0	—	—	—	—
	0.30	0.291	0.307	97.3	102
	0.50	0.493	0.516	98.6	103

## 4. Conclusion

In summary, nanostructured CB was used for electrochemical determination of Pb(II) and Cd(II) simultaneously for the first time and the fabricated electrochemical sensor showed good properties, such as lower detection limit, higher sensitivity, wider linear range, better stability and repeatability. Meanwhile, the fabricated sensor was used to determinate Cd(II) and Pb(II) in practical samples with satisfactory results. Therefore, the modified electrode has a very good practical application prospects.

## Supplementary Material

DPASV and calibration curves

## Data accessibility

All data used in this research including figures, tables have been reported in the paper and are freely available. This paper has no additional data.
