# Supplementary material for: Nanostructured carbon black for simultaneous electrochemical determination of trace lead and cadmium by differential pulse stripping voltammetry
Source: R Soc Open Sci. 2018 Jul 25;5(7):180282. doi: 10.1098/rsos.180282 (PMC6083681; doi:10.1098/rsos.180282)
Supplement: DPASV and calibration curves [file rsos180282supp1.docx]

Nanostructured carbon black for simultaneous electrochemical determination of trace lead and cadmium by differential pulse stripping voltammetry

Ruigang Xie^1^, Lingli Zhou^1,2^, Cuiling Lan^1^, Fangfang Fan^1^, Ruifeng Xie^3^, Hongyu Tan^4^ , Tiansheng Xie^1^ and Lingmin Zhao^1^

*1. Guangxi Colleges and Universities Key Laboratory of Regional Ecological Environment Analysis and Pollution Control of West Guangxi, College of Chemistry and Environment Engineering, Baise University，Baise 533000, P. R. China 2. Key Laboratory of New Processing Technology for Nonferrous Metals and Materials, Ministry of Education, College of Materials Science and Engineering, Guilin University of Technology, Guilin 541004, P. R. China 3. Ocean and Civil Engineering, Shanghai Jiao Tong University, Shanghai 200240, P. R. China 4.* *Guangxi Ferroalloy Co., Ltd. Laibin 546100, P. R. China*


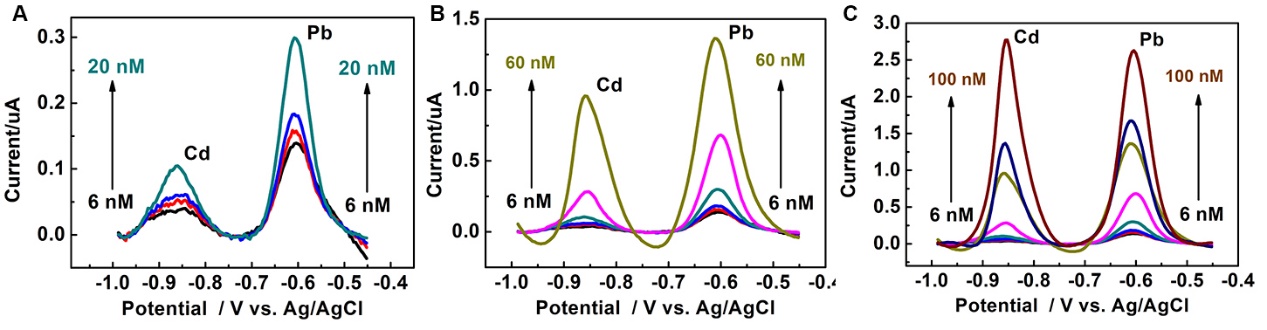


Figure S1. DPASV and calibration curves of the stripping peak currents at CB-Nafion-GCE with increased concentrations of Cd(II) and Pb(II) (A) from 6-20 nM; (B) from 6-60 nM;

(C) from 6-100 nM in 0.1 M HAc-NaAc buffer solution. Other experimental conditions were under the optimal conditions.
